# Effect of Oxygen Content on Microstructure and Tensile Properties of a 22Cr-5Al ODS Steel

**DOI:** 10.3390/ma14092241

**Published:** 2021-04-27

**Authors:** Yukun Zhang, Yingjie Yan, Yazhong Zhai, Wei Qin, Hongyan Che, Tiejun Wang, Rui Cao

**Affiliations:** 1 State Key Laboratory of Advanced Processing and Recycling of Nonferrous Metals, Lanzhou University of Technology, Langongping 287 Road, Qilihe District, Lanzhou 730050, China; zyklut@163.com (Y.Z.); zhaiyazhong1995@163.com (Y.Z.); caorui@lut.edu.cn (R.C.); 2 School of Materials Science and Engineering, Lanzhou University of Technology, Lanzhou 730050, China; 3 Advanced Technology & Materials Limited Company, China Iron & Steel Research Institute Group, Beijing 100081, China; qinwei@atmcn.com (W.Q.); chehongyan@atmcn.com (H.C.); wangtj@atmcn.com (T.W.)

**Keywords:** 22Cr-5Al ODS steel, oxygen content, Y-rich precipitates, tensile properties

## Abstract

The high tensile strength and irradiation resistance of oxide dispersion strengthened (ODS) ferritic steels is attributed to the ultrafine and dispersed oxides within the matrix. The high content of oxygen and yttrium is critical for the formation of dense Y-rich oxides. However, only few studies have reported the effect of oxygen content on the microstructure and mechanical properties of ODS steels. Herein, we employed gas atomization reactive synthesis to prepare pre-alloy powders and then hot isostatic pressing (HIP) to consolidate two 22Cr-5Al ODS steels with different oxygen content. Our results showed Y-rich precipitates at and near grain boundaries of the as-HIPed alloys. Moreover, with the oxygen content increasing from 0.04 to 0.16 wt%, more precipitates precipitated in the as-HIPed alloy, and the ultimate tensile strength of the alloy was improved. However, increasing the oxygen content to 0.16 wt% led to formation of stripe and chain precipitates at and near grain boundaries, which caused a partial intergranular fracture of the as-HIPed alloy.

## 1. Introduction

Oxide dispersion strengthened (ODS) steels are key candidate structural materials for advanced nuclear systems [1,2,3,4]. The high density of nanoscale oxides provides ODS steels with excellent mechanical performance by blocking dislocation slipping and hindering grain boundaries movement [5,6,7,8,9]. Several studies have reported that fine and thermo-stable oxides improve the creep resistance of ferritic steels [10,11,12,13] and trap He in fine bubbles, thereby limiting He movement and restraining He brittleness [2,3,4]. The common processes to fabricate ODS steels are powder metallurgy (PM) with mechanical alloying (MA) and heat consolidation [14], including hot isostatic pressure (HIP) [9], hot extrusion (HE) [15,16], and spark plasma sintering (SPS) [17], and ODS steels have a high density and an excellent tensile strength. In contrast to HIP and HE, laser additive manufacturing (LAM) [18,19] without the MA process is a method used to manufacture the ODS Fe-matrix alloys because of its low cost and flexibility [18]. Yingnan Shi et al. fabricated a Zr-containing ODS-FeCrAl alloy by LAM, which presented anisotropic tensile properties [18].

The excellent performance of ODS steels can be attributed to the rational composition design and introduction of ultrafine nano-oxides [20,21]. Previous studies have shown that micro-alloyed elements can react with Y and O to form Y-containing oxides [22,23,24,25]. Surprisingly, the addition of Al, which was expected to improve oxidation resistance of steels, deteriorated the high-temperature strength of the Al-containing ODS steel due to the coarsening tendency of Y-Al-O oxides at high temperatures [26,27]. An appropriate addition of Zr could effectively inhibit the coarsening of Al-containing oxides by forming more stable Y-Zr-O oxides in the matrix. The addition of Ti [28,29] can refine grains and form ternary oxides (Y-Ti-O), of smaller size and higher thermal stability than Y_2_O_3_, by reacting with Y and O. Improving the Ti/Y ratio can minimize the interfacial energy between particle and matrix, which reduces the coarsening rate of oxides at high temperatures [30]. The high oxygen is the main reason for the formation of powder boundaries (PPBs) in Ni-based super alloys fabricated through PM [31]. However, the high content of Y and O in ODS steel is necessary for the formation of fine and dense precipitates in the matrix to ensure its excellent comprehensive performance [32,33]. 

In this study, we prepared two Fe-22Cr-5Al ODS steels with different oxygen content in order to better understand the relationship among oxygen content, microstructure, and tensile strength. We characterized the microstructure and the precipitates of the alloys in detail, and we investigated the tensile properties and fracture behavior of the two steels at 25 °C. Finally, we discussed the relationship among oxygen, microstructure, and tensile strength according to the obtained results.

## 2. Materials and Experiment

**Figure 1:**
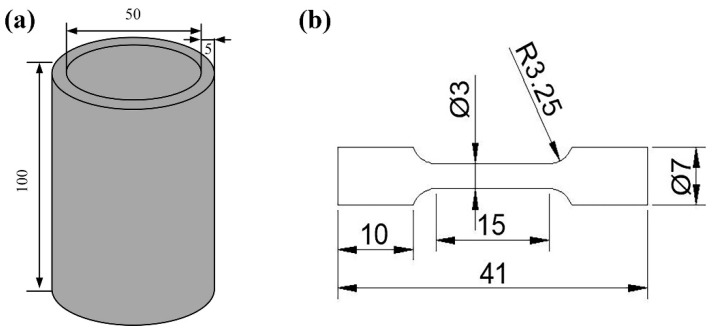
Schematic diagrams of (**a**) powder cans and (**b**) tensile samples (in mm).

We prepared Fe-22Cr-5Al-0.1Y powders with different oxygen content through Gas Atomization Reactive Synthesis (GARS) [34,35]. The powder size ranged from 50 to 350 μm. As shown in Figure 1a, the height of the powder can was 100 mm, its inner diameter was 50 mm, and the can wall thickness was 5 mm. The vacuum within the can was 10^−2^ Pa before HIP. Subsequently, the powders were consolidated by HIP at 1220 °C under a pressure of 150 MPa for 3 h [34]. The obtained alloys were named as-HIPed alloy A (oxygen content: 0.04 wt%) and B (oxygen content: 0.16 wt%), and their chemical compositions are shown in Table 1. The oxygen content of the alloys was tested by LECO TCH600 combined determination apparatus (LECO, Saint Joseph, MI, USA) for oxygen, nitrogen, and hydrogen.

Next, the phase analysis of as-HIPed alloys was carried out by X-ray Diffraction (XRD) using a D/max-2400 X-ray diffractometer (Rigaku, Tokyo, Japan) with Cu Kα, and the scan rate was 10°· min^−1^. We observed the microstructure of the as-HIPed samples using a Scanning Electron Microscope (SEM, Quanta 450FEG, FEI, Hillsboro, OR, USA) with an Energy Dispersion Spectrum (EDS) analyzer. Firstly, the samples used for SEM analysis were electro-etched in a 10% perchloric acid alcohol solution at 23 V for 30 s, followed by pickling in a 60% hydrochloric acid alcohol solution for 50 s. We then conducted Transmission Electron Microscopy (TEM, JEM-2100F, JEOL, Tokyo, Japan) analysis using thin foil samples, which were mechanically thinned to 60 μm and a diameter of 3 mm. Next, the thin foils were electropolished using an MTA-1A twin jet polisher with a solution (10% perchloric acid + 90% alcohol) at −30 °C. Finally, we carried out tensile tests at 25 °C with a strain rate of 1 mm/min. As shown in Figure 1b, the samples with a gauge length of 15 mm and a gauge diameter of 3 mm were used for the tensile strength testing by the testing machine AGS-X300 (Shimadzu, Kyoto, Japan). Three tensile samples of each alloy were tested. Fractography and EDS on the fracture surfaces were analyzed by an SEM.

## 3. Results

### 3.1. Microstructure

**Figure 2:**
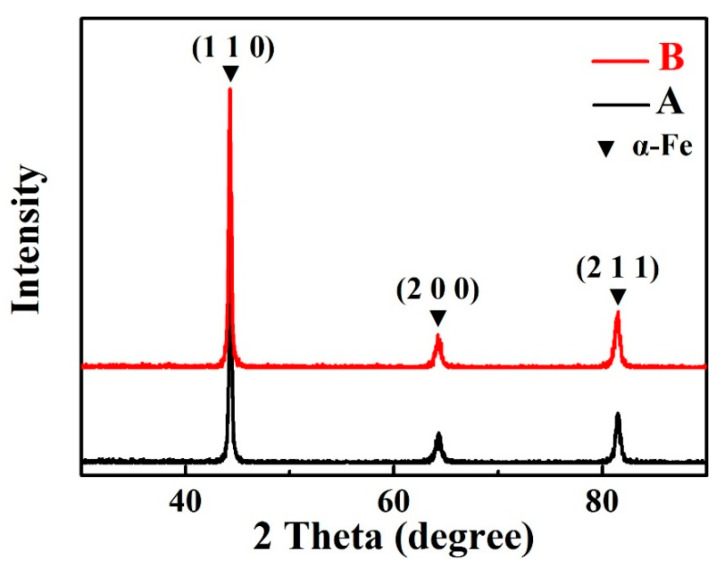
The XRD patterns of the as-HIPed alloys A and B.

**Figure 3:**
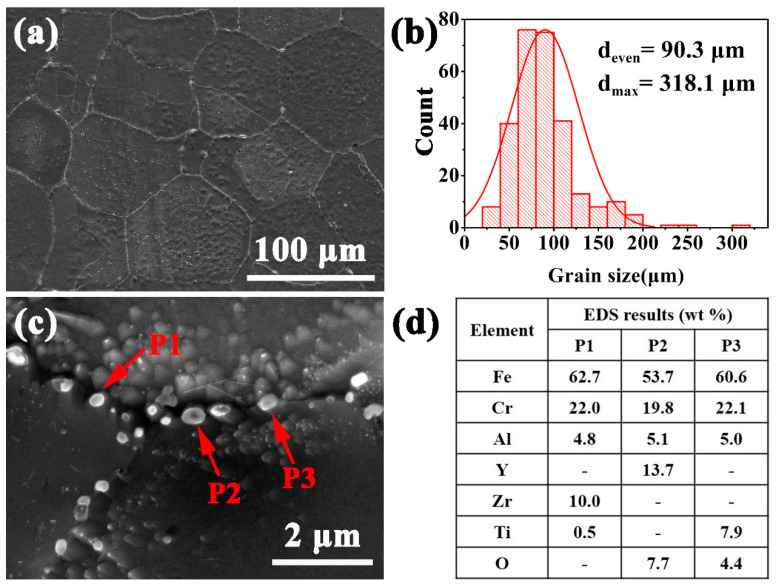
Microstructure of the as-HIPed alloy A: (**a**) SEM secondary electron micrograph of the macrostructure; (**b**) distribution of grain sizes for the as-HIPed alloy A; (**c**) SEM secondary electron micrograph of precipitates at grain boundaries and (**d**) EDS results of the precipitates in (**c**).

Figure 2 shows the XRD profiles of the as-HIPed alloys A and B. It can be seen that the matrixes of the as-HIPed A and B mainly were in the α-Fe phase due to the high Cr content [36]. Figure 3a,b shows an SEM secondary electron image of the macrostructure and the statistical distribution of grain sizes of the as-HIPed alloy A. The grain size of the alloy was calculated from the average lengths of the long and the short axes of the grains. According to the statistical analysis, the average and maximum grain sizes of the as-HIPed alloy A were 90 μm and 318 μm, respectively (Figure 3b). Figure 3c shows the microstructures of the as-HIPed alloy A. Results indicated that the as-HIPed alloy A had spherical and dispersed precipitates (pointed by red arrows), most of which were distributed at grain boundaries (Figure 3c). According to the EDS results, the precipitates at grain boundaries were Y-, Zr- and Ti-oxides (Figure 3d). 

**Figure 4:**
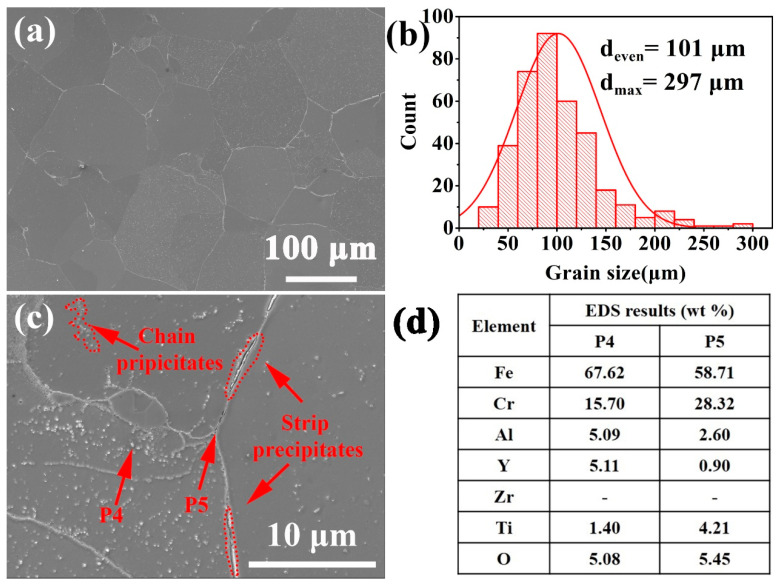
Microstructure of the as-HIPed alloy B: (**a**) SEM secondary electron micrograph of the macrostructure; (**b**) distribution of grain sizes for the as-HIPed alloy B; (**c**) SEM secondary electron micrograph of precipitates at grain boundaries and (**d**) EDS results of the precipitates in (**c**).

Figure 4a,b shows an SEM secondary electron image of the macrostructure and the statistical distribution of grain sizes of the as-HIPed alloy B. The average and maximum grain sizes of the as-HIPed alloy B were 101 μm and 297 μm, respectively (Figure 4b). Figure 4c,d shows the microstructure of the as-HIPed alloy B and the EDS results of the precipitates pointed in Figure 4c. Results showed that in the as-HIPed alloy B, there were not only white spherical Y- and Ti-rich oxides at grain boundaries and within grains, but also stripe and chain precipitates at and near grain boundaries (highlighted by a red dashed line). 

**Figure 5:**
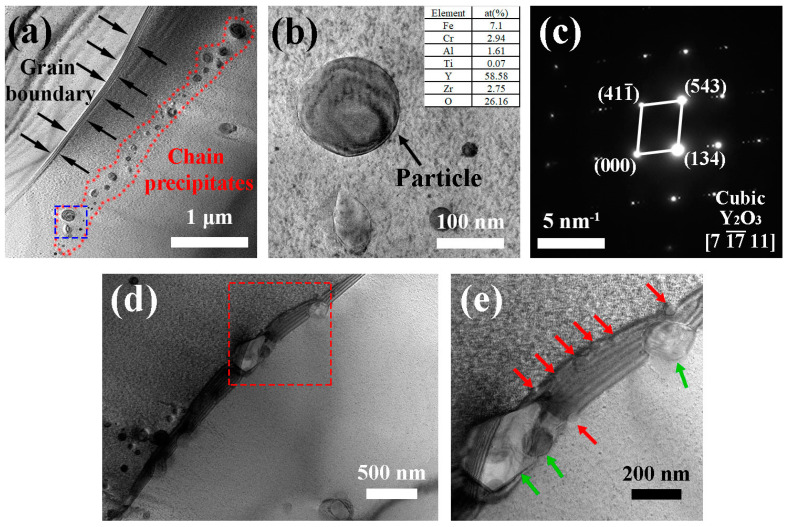
(**a**) TEM bright field image of continuous precipitates near the grain boundary in the as-HIPed alloy B; (**b**) high magnification of the area inside the dashed blue line in (**a**); (**c**) SAD pattern for the arrowed particle in (**b**); (**d**) TEM bright field image of the precipitates at the grain boundary; (**e**) high magnification of the area inside the dashed red line in (**d**); grain boundary precipitates of different sizes are pointed by red and green arrows.

We also conducted TEM characterizations in order to systematically investigate the chain/stripe precipitates found in the as-HIPed alloy B. Figure 5 shows bright field images of precipitates in the as-HIPed alloy B. Results showed that there was a chain of precipitates with sizes ranging from 30 to 230 nm (marked by a red dashed line) close to the grain boundary (marked by black arrows) (Figure 5a). Figure 5b shows a magnified image of the precipitates in Figure 5a marked by a blue dashed line. Moreover, the EDS results (Figure 5b) indicated that the spherical precipitates (diameter about 150 nm, pointed by black arrows) had high Y and O content, while SAD (selective area diffraction) patterns confirmed that the precipitate (Figure 5c) was cubic Y_2_O_3_ with a space group Ia-3 (206) with a = b = c = 1.062 nm and α = β = γ = 90°.

Continuous precipitates were found at the grain boundaries (Figure 5d). Figure 5e is a magnification of the zone in Figure 5d marked by a red dotted line. The size of the precipitates in Figure 5e close to and at grain boundaries varied from 20 to 500 nm. Furthermore, small-sized precipitates (about 20 nm, pointed by red arrows) were distributed at grain boundaries and around large-sized precipitates (over 200 nm, pointed by green arrows), thereby forming a stripe at grain boundaries.

### 3.2. Tensile Test and Fracture Surface Analysis

**Figure 6:**
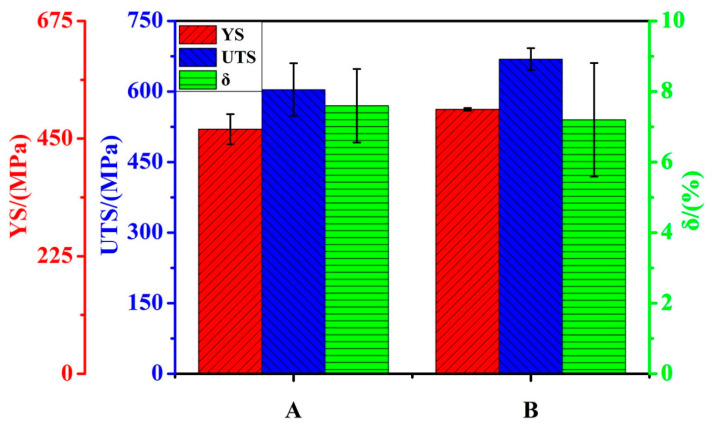
The ultimate tensile strength (UTS), yield strength (YS), and elongation (δ) for the as-HIPed alloys A and B at 25 °C.

We determined the tensile strengths of both as-HIPed alloys at 25 °C. Figure 6 shows their yield strength (YS), ultimate tensile strength (UTS), and elongation (A). The average UTSs of the as-HIPed alloys A and B were 604 MPa and 669 MPa, respectively. On the other hand, the average YS of the as-HIPed alloy A was 468 MPa and that of the as-HIPed alloy B was 506 MPa. In addition, both samples had a brittle facture during the tensile test at 25 °C. Our results indicated that the fracture elongations of the as-HIPed A and B were 7.6% and 7.2%, respectively. Afterwards, the SEM was used for fractography on the tensile tested samples in order to understand the effect of the precipitates on the fracture behavior of these as-HIPed alloys.

**Figure 7:**
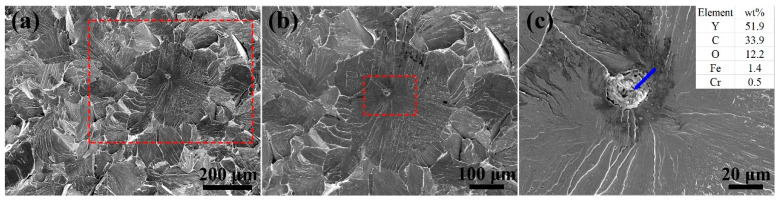
SEM secondary electron micrographs of the fractography for the tensile tested sample: (**a**) macro-morphology; (**b**,**c**) micrographs of the crack source surface of the as-HIPed alloy A at a high magnification.

Figure 7 shows the fractography of the as-HIPed alloy A. Results showed a brittle fracture in the as-HIPed alloy A, and the fracture surface was a cleavage fracture (Figure 7a). Furthermore, we observed a large aggregated particle (about 22 μm) at the fracture source (marked by a red dashed frame in Figure 7b and a blue arrow in Figure 7c) along the river pattern at the fracture surface. The magnified image of the particle and the EDS results are shown in Figure 7c. These EDS results indicated that the aggregated particle was Y-rich. In addition, there was a high C content in the aggregated Y-rich oxide. The high C content of the large aggregated particles may come from two sources: one is organisms deposited on the powder surfaces during the process of materials preparation; another is residual impurities, such as carbide or dust, which were introduced during the preparation and collection of the powders. The aggregated oxide particles were observed on two of the fracture surfaces of the three tested samples. Although the aggregated oxide particles are not a major issue, they are a reason for the instability of the tensile strength and should be avoided.

**Figure 8:**
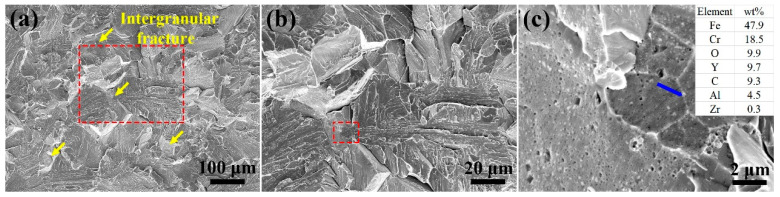
SEM secondary electron micrographs of the fractography for the tensile tested sample: (**a**) macro-morphology; (**b**,**c**) micrographs of the crack source surface of the as-HIPed alloy B at a high magnification.

Figure 8 shows the fractography of the as-HIPed alloy B. The magnified image of the area marked by a red dashed frame in Figure 8a is shown in Figure 8b. The tensile test results revealed that there was also a brittle fracture in the as-HIPed alloy B (Figure 8a). The fracture surface was mainly a cleavage fracture, with a partial area having an intergranular fracture, which is pointed by yellow arrows in Figure 8a. An intergranular fracture was observed on the fracture surfaces of all three tested samples. The EDS results indicated that there were fine and dense Y-rich precipitates on the fracture surface (Figure 8c), which were the main reason for the occurrence of a partial intergranular fracture in the as-HIPed alloy B.

## 4. Discussion

The microstructure and mechanical properties of powder metallurgy alloys are influenced by the oxygen content of powders and oxides on the powder surface [31,34,37]. During the gas atomization process, the atomized powders would inevitably react with oxygen in the environment to form metastable Fe- and Cr-oxides on the powder surface [38]. Subsequently, the metastable oxides would decompose and react with the Y element during hot isostatic pressing, which segregated at grain boundaries due to the low solid solution in the Fe-based alloy at high temperatures, thereby forming fine and stable Y-containing precipitates at and near grain boundaries [39,40,41], as shown in Figure 3c and Figure 4c. During this process, the metastable Fe- and Cr-oxides on the powder surface would provide a nucleation position and partial oxygen to promote the formation of stable Y-containing precipitates at and near grain boundaries. 

The excellent mechanical property of ODS steels is attributed to the fine and stable dispersed oxides. Li et al. reported that the average size of nano-oxides in 16Cr ODS steel slightly increased with a percentage of 15.7% when the aging time was up to 5000 h at 973 K [21]. Increasing the oxygen content and adding Y and Zr elements can promote the formation of thermodynamically stable nano-oxides [25,42]. Our results indicated that in the as-HIPed alloy A, the fine and stable Y-rich oxides were mainly distributed at and near grain boundaries. Moreover, increasing the oxygen content from 0.04 to 0.16 wt% led to the precipitation of a large amount of fine Y-rich oxides within grains in the as-HIPed alloy B, and formation of chain oxides near and at grain boundaries. Even the small spherical Y_2_O_3_ oxides grew into stripe oxides at grain boundaries. In addition, Y could react with O and Ti/Al/Zr to form more stable ternary Y-Ti/Al/Zr-O oxides (Y_2_Ti_2_O_7_, Y_2_TiO_5_, Y_3_Al_5_O_12_, Y_4_Al_2_O_9_, YAlO_3_, Y_4_Zr_3_O_12_) [8,26,27,28,29]. The formation of these ternary Y-containing oxides needs to consume more oxygen compared to the formation of Y_2_O_3_. 

The dispersed precipitates at grain boundaries and in the matrix are the main reasons for the high tensile and creep strength of ODS steels [12,20]. With a precipitate density increase and a size decrease, the yield strength and tensile strength of ODS steels increased, but the elongation decreased and a brittle fracture occurred [33]. The fine and dispersed oxides improved the tensile strength of alloys by pinning dislocations and blocking grain boundary movement [5]. The results showed that increasing the oxygen content increased the ultimate tensile strength and yield strength of the as-HIPed alloy B by 10.7% and 8.1%, respectively, compared to the as-HIPed alloy A [43]. A brittle fracture occurred in both as-HIPed alloys A and B, and the mean elongations of the two alloys were 7.6% and 7.2%, respectively. The brittle fracture of both alloys could be attributed to the aggregated large-sized Y-rich particle and the stripe/chain precipitates near and at grain boundaries, which could decrease the strength of grain boundaries. They easily become crack initiations due to stress concentration, thereby causing a brittle fracture of alloys during a tensile test [44]. 

Notably, compared to the FeCrAl-ODS steels prepared by MA following HIP with grain sizes of less than 10 μm [45,46], the strength of the alloys in this study was low. There are three factors responsible for the low strength of as-HIPed alloys: large grain size, numerous impure particles in the matrix, and low density of the precipitates within grains. The grain size of about 100 µm is larger than in other ODS alloys prepared by mechanical alloying [25,28]. The larger grain size is detrimental to the toughness of the alloys. Refining the grain size can improve the toughness and strength of an alloy. Lowering HIP temperature and shortening HIP time can prevent the grain growth during the HIP process [34]. Optimizing the composition of powders, reducing the as-atomized powder size, and avoiding the impurity particles in powders can effectively improve the strength and ductility of an alloy [32,47]. Furthermore, the grain size can be refined by thermomechanical processing after HIP [28,43]. The large and continuous precipitates in the alloy can also be refined and dispersed during the thermomechanical process, which may be an effective method for improving the strength and toughness of as-HIPed alloys.

## 5. Conclusions

In this study, we produced two 22Cr-5Al ODS steels with different oxygen contents through the GARS route and HIP. We then conducted microstructure characterization and tensile tests at room temperature in order to investigate the effect of oxygen content on the microstructure and tensile strength of the as-HIPed 22Cr-5Al ODS steels. The following conclusions were drawn:

1. The precipitates in the as-HIPed alloys mainly were Y-rich oxides. An increase in the oxygen content from 0.04 to 0.16 wt% led to an increase in the number and density of precipitates in the as-HIPed alloy. Meanwhile, the Y-rich precipitates segregated at and near grain boundaries and formed large-sized stripe/chain precipitates.
2. With the increase in oxygen content from 0.04 to 0.16 wt%, the ultimate tensile strength of the as-HIPed alloy increased from 604 to 669 MPa, and the yield strength increased from 468 to 506 MPa. However, a brittle fracture occurred in both as-HIPed alloys, which could be attributed to the segregation of large-sized oxides and the stripe/chain Y-rich precipitates at and near grain boundaries.

## Figures and Tables

**Table 1:** Chemical composition of the as-HIPed ODS steels (wt%).

	O	Cr	Al	Ti	Y	Zr	Fe
A	0.04	22	5	0.11	0.11	0.1	Bal.
B	0.16	22	5	0.12	0.10	0.1	Bal.

## Data Availability

The data presented in this study are available on request from the corresponding author.
